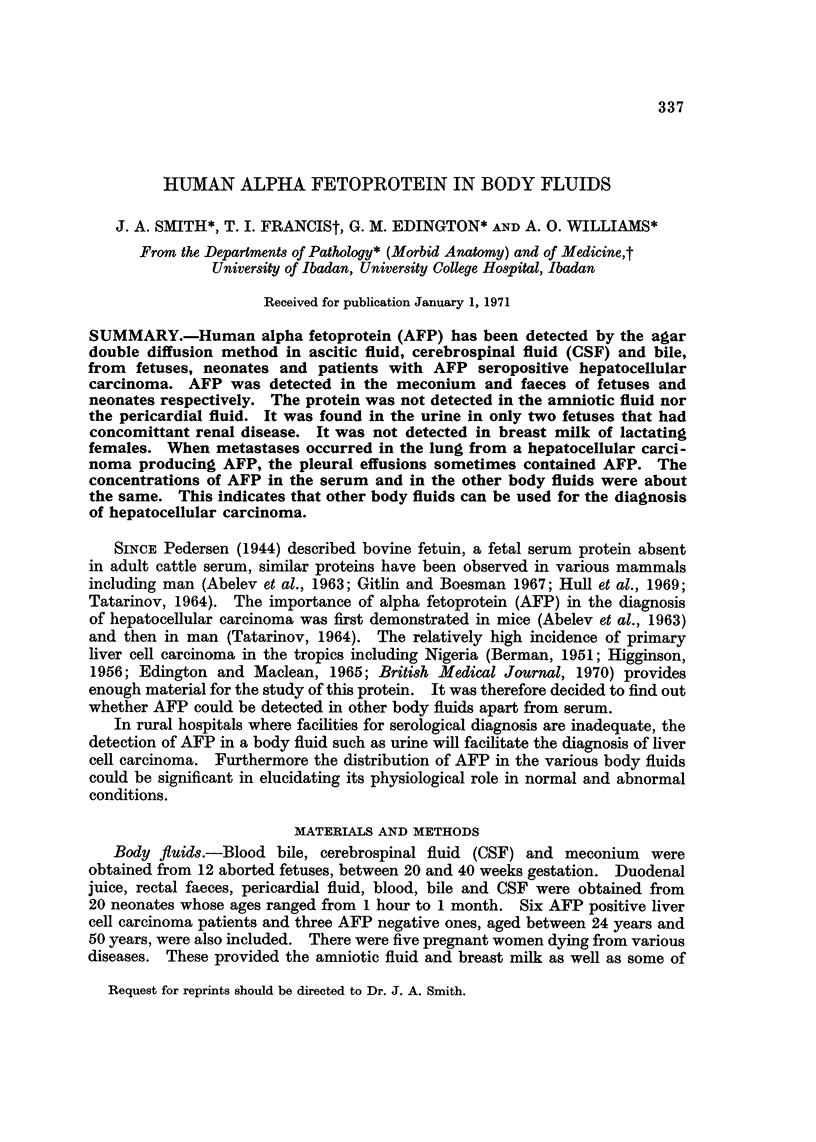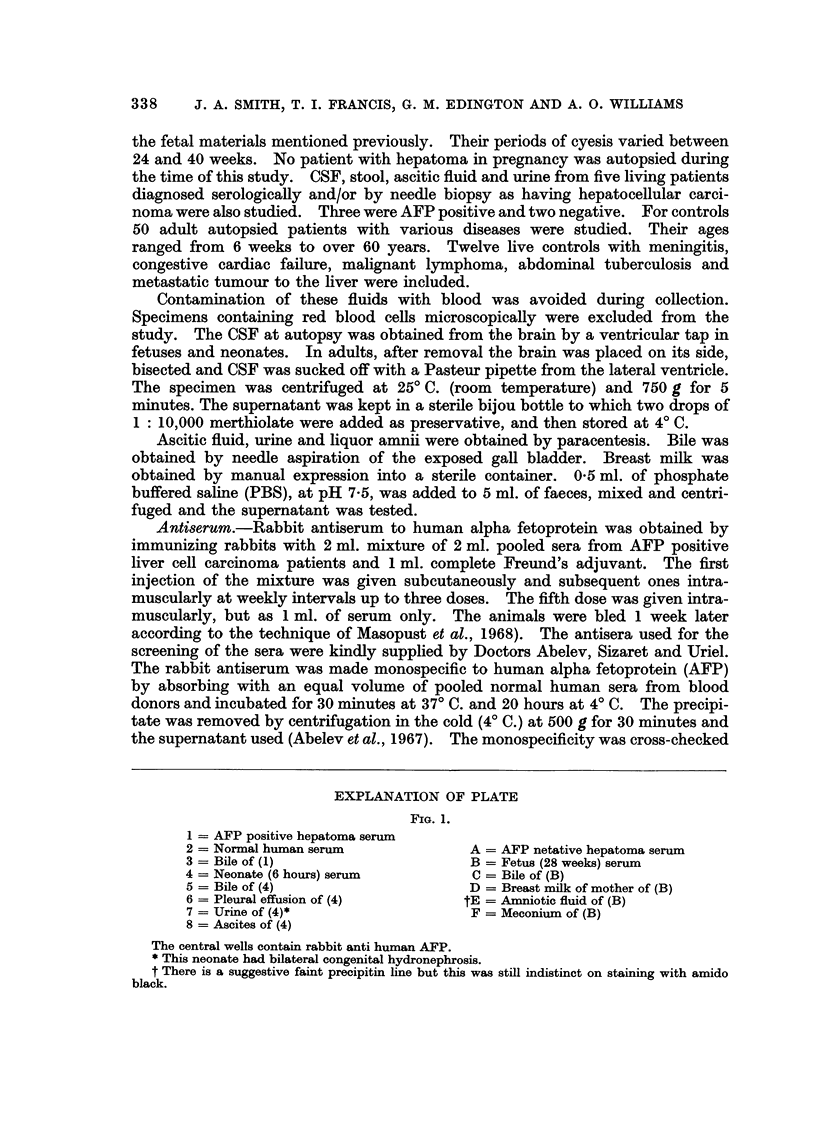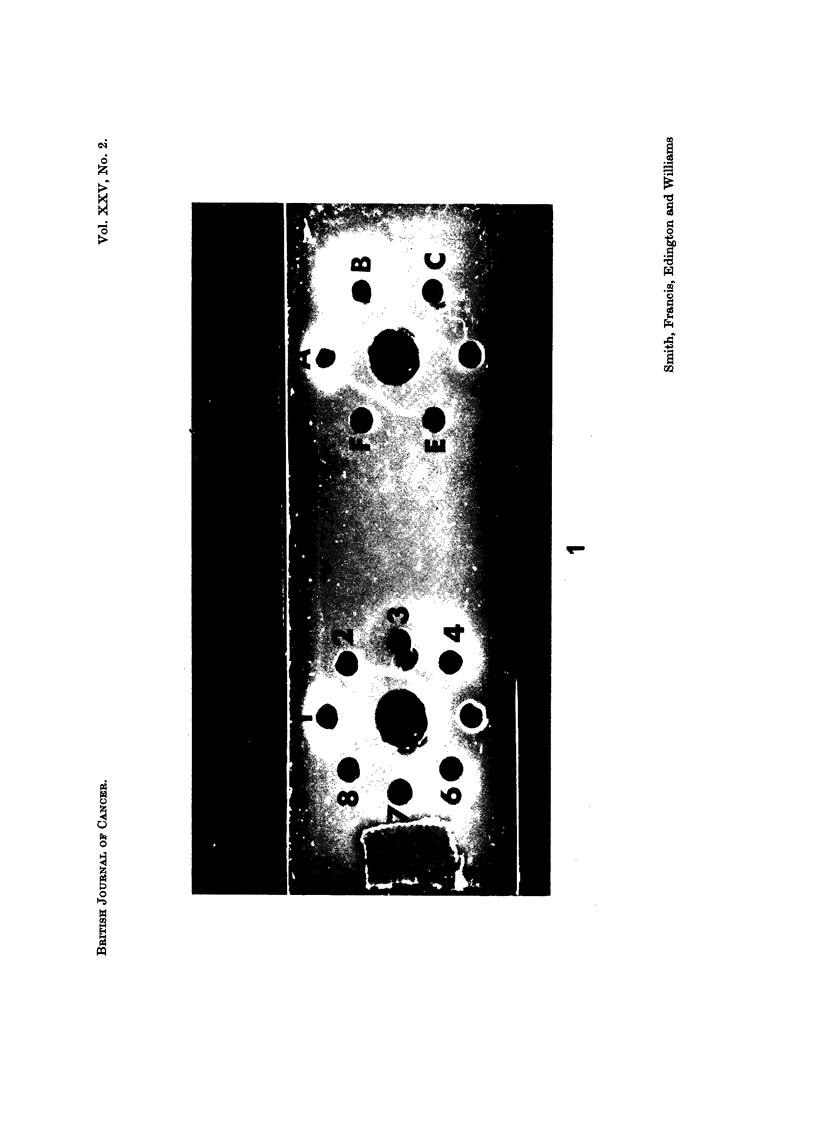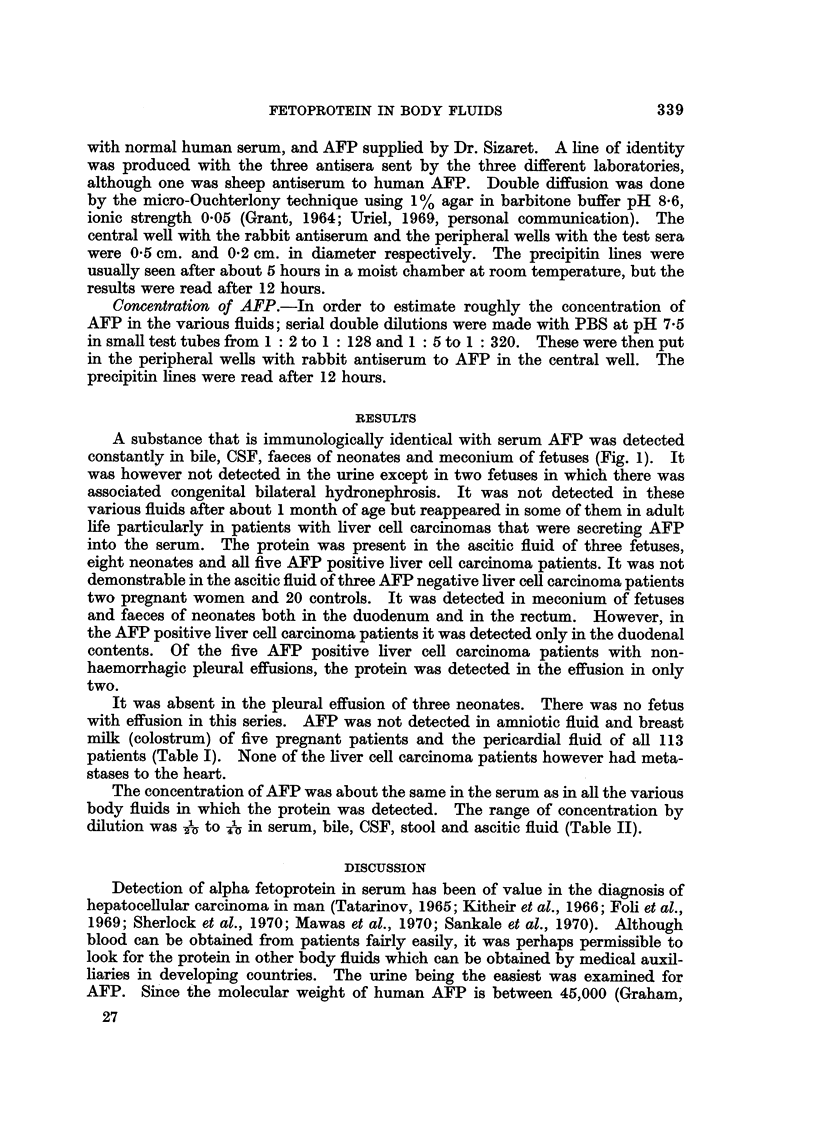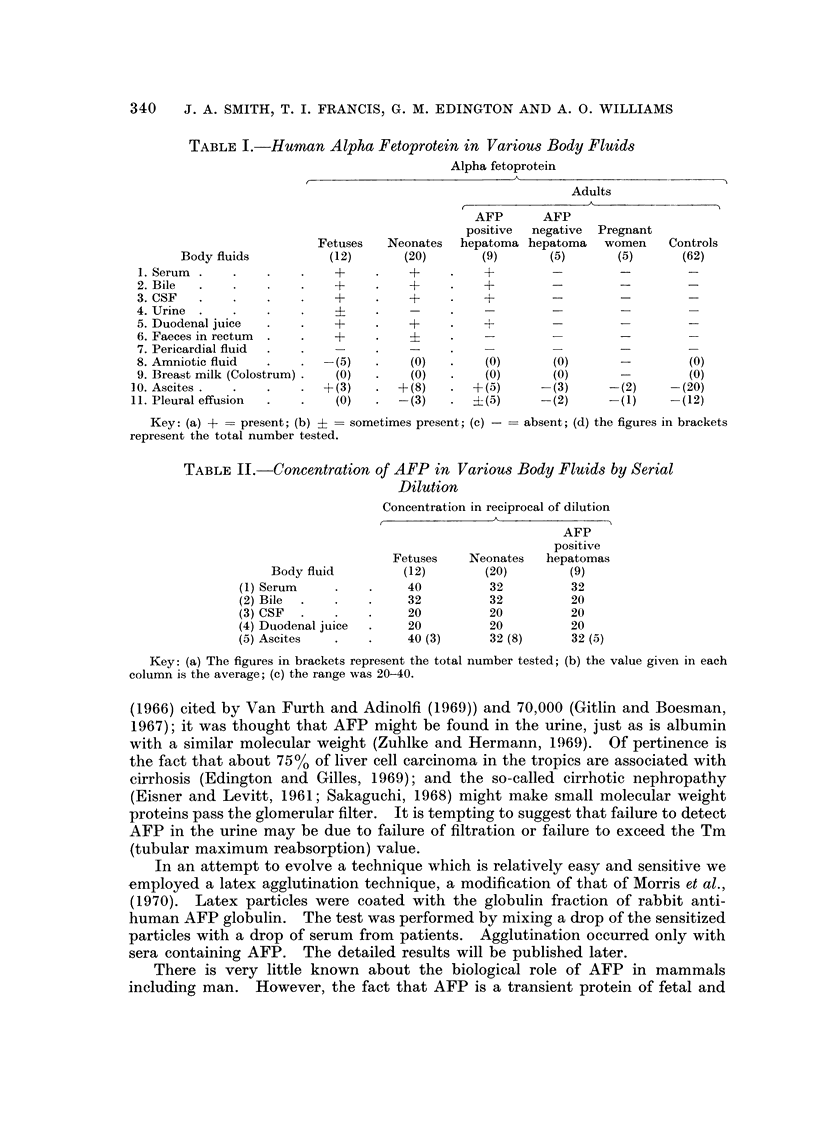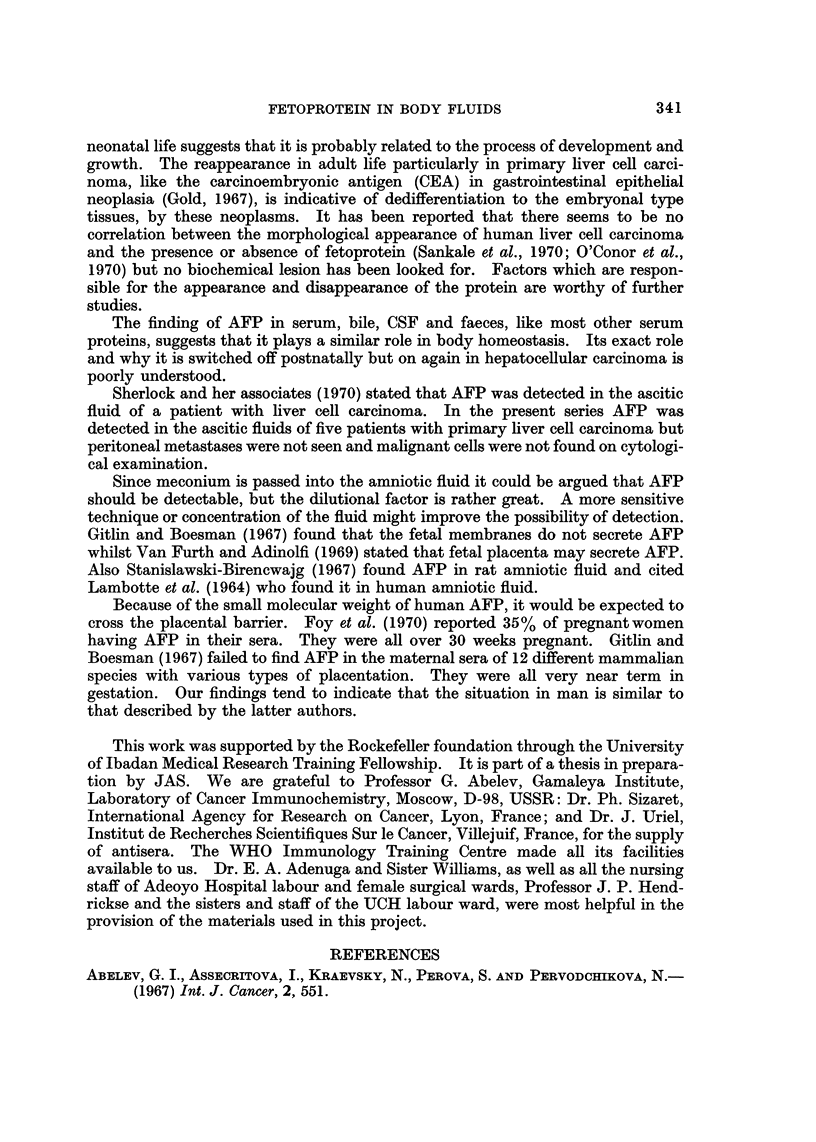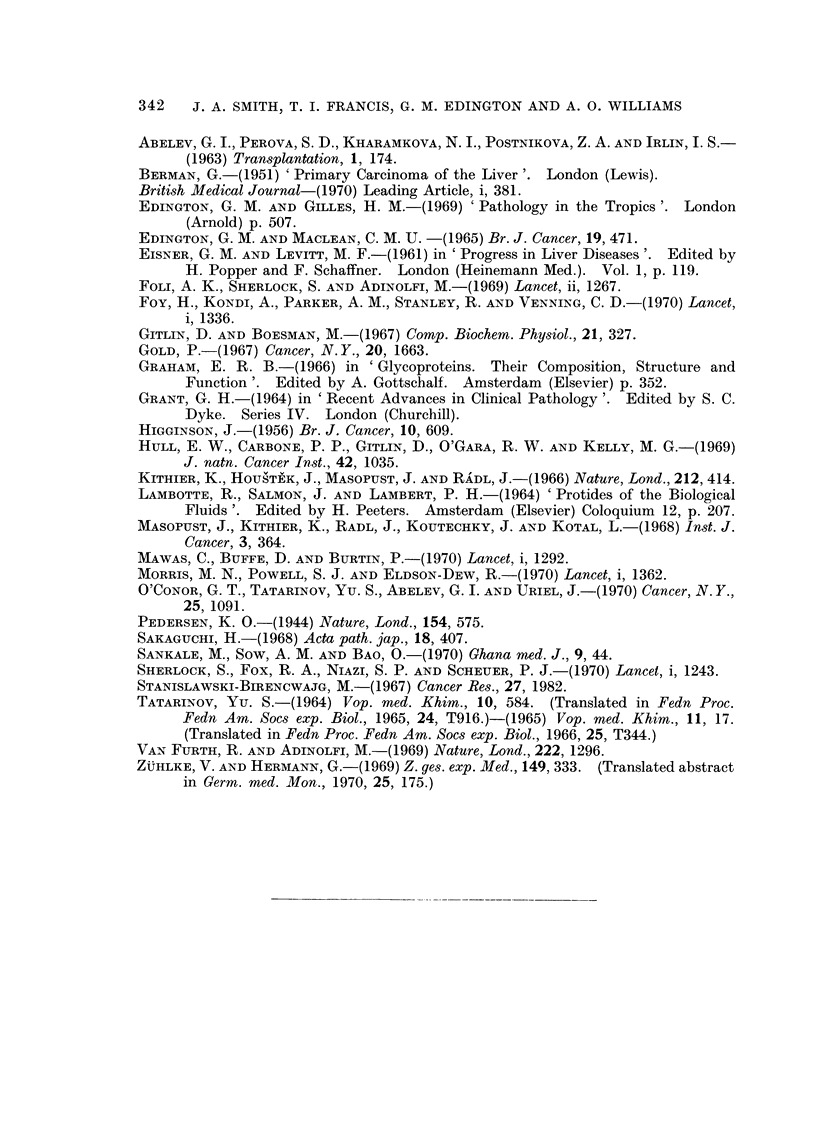# Human Alpha Fetoprotein in Body Fluids

**DOI:** 10.1038/bjc.1971.43

**Published:** 1971-06

**Authors:** J. A. Smith, T. I. Francis, G. M. Edington, A. O. Williams

## Abstract

**Images:**


### HUMAN ALPHA FETOPROTEIN IN BODY FLUIDS

J. A. SMITH*, T. I. FRANCIS†, G. M. EDINGTON* AND A. O. WILLIAMS*

*From the Departments of Pathology* (Morbid Anatomy) and of Medicine,†*
*University of Ibadan, University College Hospital, Ibadan*

Received for publication January 1, 1971

**SUMMARY.—Human alpha fetoprotein (AFP) has been detected by the agar double diffusion method in ascitic fluid, cerebrospinal fluid (CSF) and bile, from fetuses, neonates and patients with AFP seropositive hepatocellular carcinoma. AFP was detected in the meconium and faeces of fetuses and neonates respectively. The protein was not detected in the amniotic fluid nor the pericardial fluid. It was found in the urine in only two fetuses that had concomittant renal disease. It was not detected in breast milk of lactating females. When metastases occurred in the lung from a hepatocellular carcinoma producing AFP, the pleural effusions sometimes contained AFP. The concentrations of AFP in the serum and in the other body fluids were about the same. This indicates that other body fluids can be used for the diagnosis of hepatocellular carcinoma.**

SINCE Pedersen (1944) described bovine fetuin, a fetal serum protein absent in adult cattle serum, similar proteins have been observed in various mammals including man (Abelev *et al.*, 1963; Gitlin and Boesman 1967; Hull *et al.*, 1969; Tatarinov, 1964). The importance of alpha fetoprotein (AFP) in the diagnosis of hepatocellular carcinoma was first demonstrated in mice (Abelev *et al.*, 1963) and then in man (Tatarinov, 1964). The relatively high incidence of primary liver cell carcinoma in the tropics including Nigeria (Berman, 1951; Higginson, 1956; Edington and Maclean, 1965; *British Medical Journal*, 1970) provides enough material for the study of this protein. It was therefore decided to find out whether AFP could be detected in other body fluids apart from serum.

In rural hospitals where facilities for serological diagnosis are inadequate, the detection of AFP in a body fluid such as urine will facilitate the diagnosis of liver cell carcinoma. Furthermore the distribution of AFP in the various body fluids could be significant in elucidating its physiological role in normal and abnormal conditions.

### MATERIALS AND METHODS

*Body fluids.*—Blood bile, cerebrospinal fluid (CSF) and meconium were obtained from 12 aborted fetuses, between 20 and 40 weeks gestation. Duodenal juice, rectal faeces, pericardial fluid, blood, bile and CSF were obtained from 20 neonates whose ages ranged from 1 hour to 1 month. Six AFP positive liver cell carcinoma patients and three AFP negative ones, aged between 24 years and 50 years, were also included. There were five pregnant women dying from various diseases. These provided the amniotic fluid and breast milk as well as some of

Request for reprints should be directed to Dr. J. A. Smith.

the fetal materials mentioned previously. Their periods of cyesis varied between 24 and 40 weeks. No patient with hepatoma in pregnancy was autopsied during the time of this study. CSF, stool, ascitic fluid and urine from five living patients diagnosed serologically and/or by needle biopsy as having hepatocellular carcinoma were also studied. Three were AFP positive and two negative. For controls 50 adult autopsied patients with various diseases were studied. Their ages ranged from 6 weeks to over 60 years. Twelve live controls with meningitis, congestive cardiac failure, malignant lymphoma, abdominal tuberculosis and metastatic tumour to the liver were included.

Contamination of these fluids with blood was avoided during collection. Specimens containing red blood cells microscopically were excluded from the study. The CSF at autopsy was obtained from the brain by a ventricular tap in fetuses and neonates. In adults, after removal the brain was placed on its side, bisected and CSF was sucked off with a Pasteur pipette from the lateral ventricle. The specimen was centrifuged at 25° C. (room temperature) and 750 *g* for 5 minutes. The supernatant was kept in a sterile bijou bottle to which two drops of 1 : 10,000 merthiolate were added as preservative, and then stored at 4° C.

Ascitic fluid, urine and liquor amnii were obtained by paracentesis. Bile was obtained by needle aspiration of the exposed gall bladder. Breast milk was obtained by manual expression into a sterile container. 0·5 ml. of phosphate buffered saline (PBS), at pH 7·5, was added to 5 ml. of faeces, mixed and centrifuged and the supernatant was tested.

*Antiserum.*—Rabbit antiserum to human alpha fetoprotein was obtained by immunizing rabbits with 2 ml. mixture of 2 ml. pooled sera from AFP positive liver cell carcinoma patients and 1 ml. complete Freund's adjuvant. The first injection of the mixture was given subcutaneously and subsequent ones intramuscularly at weekly intervals up to three doses. The fifth dose was given intramuscularly, but as 1 ml. of serum only. The animals were bled 1 week later according to the technique of Masopust *et al.*, 1968). The antisera used for the screening of the sera were kindly supplied by Doctors Abelev, Sizaret and Uriel. The rabbit antiserum was made monospecific to human alpha fetoprotein (AFP) by absorbing with an equal volume of pooled normal human sera from blood donors and incubated for 30 minutes at 37° C. and 20 hours at 4° C. The precipitate was removed by centrifugation in the cold (4° C.) at 500 *g* for 30 minutes and the supernatant used (Abelev *et al.*, 1967). The monospecificity was cross-checked

---

EXPLANATION OF PLATE

FIG. 1.

1 = AFP positive hepatoma serum

2 = Normal human serum

3 = Bile of (1)

4 = Neonate (6 hours) serum

5 = Bile of (4)

6 = Pleural effusion of (4)

7 = Urine of (4)*

8 = Ascites of (4)

A = AFP netative hepatoma serum

B = Fetus (28 weeks) serum

C = Bile of (B)

D = Breast milk of mother of (B)

†E = Amniotic fluid of (B)

F = Meconium of (B)

The central wells contain rabbit anti human AFP.

* This neonate had bilateral congenital hydronephrosis.

† There is a suggestive faint precipitin line but this was still indistinct on staining with amido black.

1

Smith, Francis, Edington and Williams with normal human serum, and AFP supplied by Dr. Sizaret. A line of identity was produced with the three antisera sent by the three different laboratories, although one was sheep antiserum to human AFP. Double diffusion was done by the micro-Ouchterlony technique using 1% agar in barbitone buffer pH 8·6, ionic strength 0·05 (Grant, 1964; Uriel, 1969, personal communication). The central well with the rabbit antiserum and the peripheral wells with the test sera were 0·5 cm. and 0·2 cm. in diameter respectively. The precipitin lines were usually seen after about 5 hours in a moist chamber at room temperature, but the results were read after 12 hours.

*Concentration of AFP.*—In order to estimate roughly the concentration of AFP in the various fluids; serial double dilutions were made with PBS at pH 7·5 in small test tubes from 1 : 2 to 1 : 128 and 1 : 5 to 1 : 320. These were then put in the peripheral wells with rabbit antiserum to AFP in the central well. The precipitin lines were read after 12 hours.

### RESULTS

A substance that is immunologically identical with serum AFP was detected constantly in bile, CSF, faeces of neonates and meconium of fetuses (Fig. 1). It was however not detected in the urine except in two fetuses in which there was associated congenital bilateral hydronephrosis. It was not detected in these various fluids after about 1 month of age but reappeared in some of them in adult life particularly in patients with liver cell carcinomas that were secreting AFP into the serum. The protein was present in the ascitic fluid of three fetuses, eight neonates and all five AFP positive liver cell carcinoma patients. It was not demonstrable in the ascitic fluid of three AFP negative liver cell carcinoma patients two pregnant women and 20 controls. It was detected in meconium of fetuses and faeces of neonates both in the duodenum and in the rectum. However, in the AFP positive liver cell carcinoma patients it was detected only in the duodenal contents. Of the five AFP positive liver cell carcinoma patients with non-haemorrhagic pleural effusions, the protein was detected in the effusion in only two.

It was absent in the pleural effusion of three neonates. There was no fetus with effusion in this series. AFP was not detected in amniotic fluid and breast milk (colostrum) of five pregnant patients and the pericardial fluid of all 113 patients (Table I). None of the liver cell carcinoma patients however had metastases to the heart.

The concentration of AFP was about the same in the serum as in all the various body fluids in which the protein was detected. The range of concentration by dilution was $\frac{1}{20}$ to $\frac{1}{40}$ in serum, bile, CSF, stool and ascitic fluid (Table II).

### DISCUSSION

Detection of alpha fetoprotein in serum has been of value in the diagnosis of hepatocellular carcinoma in man (Tatarinov, 1965; Kitheir *et al.*, 1966; Foli *et al.*, 1969; Sherlock *et al.*, 1970; Mawas *et al.*, 1970; Sankale *et al.*, 1970). Although blood can be obtained from patients fairly easily, it was perhaps permissible to look for the protein in other body fluids which can be obtained by medical auxilliaries in developing countries. The urine being the easiest was examined for AFP. Since the molecular weight of human AFP is between 45,000 (Graham,

TABLE I.—*Human Alpha Fetoprotein in Various Body Fluids*

| | Alpha fetoprotein | | | | | |
|---|---|---|---|---|---|---|
| | | | Adults | | | |
| Body fluids | Fetuses (12) | Neonates (20) | AFP positive hepatoma (9) | AFP negative hepatoma (5) | Pregnant women (5) | Controls (62) |
| 1. Serum | + | + | + | − | − | − |
| 2. Bile | + | + | + | − | − | − |
| 3. CSF | + | + | + | − | − | − |
| 4. Urine | ± | − | − | − | − | − |
| 5. Duodenal juice | + | + | + | − | − | − |
| 6. Faeces in rectum | + | ± | − | − | − | − |
| 7. Pericardial fluid | − | − | − | − | − | − |
| 8. Amniotic fluid | −(5) | (0) | (0) | (0) | − | (0) |
| 9. Breast milk (Colostrum) | (0) | (0) | (0) | (0) | − | (0) |
| 10. Ascites | +(3) | +(8) | +(5) | −(3) | −(2) | −(20) |
| 11. Pleural effusion | (0) | −(3) | ±(5) | −(2) | −(1) | −(12) |

Key: (a) + = present; (b) ± = sometimes present; (c) − = absent; (d) the figures in brackets represent the total number tested.

TABLE II.—*Concentration of AFP in Various Body Fluids by Serial Dilution*

| | Concentration in reciprocal of dilution | | |
|---|---|---|---|
| Body fluid | Fetuses (12) | Neonates (20) | AFP positive hepatomas (9) |
| (1) Serum | 40 | 32 | 32 |
| (2) Bile | 32 | 32 | 20 |
| (3) CSF | 20 | 20 | 20 |
| (4) Duodenal juice | 20 | 20 | 20 |
| (5) Ascites | 40 (3) | 32 (8) | 32 (5) |

Key: (a) The figures in brackets represent the total number tested; (b) the value given in each column is the average; (c) the range was 20–40.

(1966) cited by Van Furth and Adinolfi (1969)) and 70,000 (Gitlin and Boesman, 1967); it was thought that AFP might be found in the urine, just as is albumin with a similar molecular weight (Zuhlke and Hermann, 1969). Of pertinence is the fact that about 75% of liver cell carcinoma in the tropics are associated with cirrhosis (Edington and Gilles, 1969); and the so-called cirrhotic nephropathy (Eisner and Levitt, 1961; Sakaguchi, 1968) might make small molecular weight proteins pass the glomerular filter. It is tempting to suggest that failure to detect AFP in the urine may be due to failure of filtration or failure to exceed the Tm (tubular maximum reabsorption) value.

In an attempt to evolve a technique which is relatively easy and sensitive we employed a latex agglutination technique, a modification of that of Morris *et al.*, (1970). Latex particles were coated with the globulin fraction of rabbit anti-human AFP globulin. The test was performed by mixing a drop of the sensitized particles with a drop of serum from patients. Agglutination occurred only with sera containing AFP. The detailed results will be published later.

There is very little known about the biological role of AFP in mammals including man. However, the fact that AFP is a transient protein of fetal and neonatal life suggests that it is probably related to the process of development and growth. The reappearance in adult life particularly in primary liver cell carcinoma, like the carcinoembryonic antigen (CEA) in gastrointestinal epithelial neoplasia (Gold, 1967), is indicative of dedifferentiation to the embryonal type tissues, by these neoplasms. It has been reported that there seems to be no correlation between the morphological appearance of human liver cell carcinoma and the presence or absence of fetoprotein (Sankale *et al.*, 1970; O'Conor *et al.*, 1970) but no biochemical lesion has been looked for. Factors which are responsible for the appearance and disappearance of the protein are worthy of further studies.

The finding of AFP in serum, bile, CSF and faeces, like most other serum proteins, suggests that it plays a similar role in body homeostasis. Its exact role and why it is switched off postnatally but on again in hepatocellular carcinoma is poorly understood.

Sherlock and her associates (1970) stated that AFP was detected in the ascitic fluid of a patient with liver cell carcinoma. In the present series AFP was detected in the ascitic fluids of five patients with primary liver cell carcinoma but peritoneal metastases were not seen and malignant cells were not found on cytological examination.

Since meconium is passed into the amniotic fluid it could be argued that AFP should be detectable, but the dilutional factor is rather great. A more sensitive technique or concentration of the fluid might improve the possibility of detection. Gitlin and Boesman (1967) found that the fetal membranes do not secrete AFP whilst Van Furth and Adinolfi (1969) stated that fetal placenta may secrete AFP. Also Stanislawski-Birencwajg (1967) found AFP in rat amniotic fluid and cited Lambotte *et al.* (1964) who found it in human amniotic fluid.

Because of the small molecular weight of human AFP, it would be expected to cross the placental barrier. Foy *et al.* (1970) reported 35% of pregnant women having AFP in their sera. They were all over 30 weeks pregnant. Gitlin and Boesman (1967) failed to find AFP in the maternal sera of 12 different mammalian species with various types of placentation. They were all very near term in gestation. Our findings tend to indicate that the situation in man is similar to that described by the latter authors.

This work was supported by the Rockefeller foundation through the University of Ibadan Medical Research Training Fellowship. It is part of a thesis in preparation by JAS. We are grateful to Professor G. Abelev, Gamaleya Institute, Laboratory of Cancer Immunochemistry, Moscow, D-98, USSR: Dr. Ph. Sizaret, International Agency for Research on Cancer, Lyon, France; and Dr. J. Uriel, Institut de Recherches Scientifiques Sur le Cancer, Villejuif, France, for the supply of antisera. The WHO Immunology Training Centre made all its facilities available to us. Dr. E. A. Adenuga and Sister Williams, as well as all the nursing staff of Adeoyo Hospital labour and female surgical wards, Professor J. P. Hendrickse and the sisters and staff of the UCH labour ward, were most helpful in the provision of the materials used in this project.

### REFERENCES

Abelev, G. I., Assecritova, I., Kraevsky, N., Perova, S. and Pervodchikova, N.—(1967) *Int. J. Cancer*, **2**, 551.

ABELEV, G. I., PEROVA, S. D., KHARAMKOVA, N. I., POSTNIKOVA, Z. A. AND IRLIN, I. S.—(1963) *Transplantation*, **1**, 174.

BERMAN, G.—(1951) ' Primary Carcinoma of the Liver '. London (Lewis).

*British Medical Journal*—(1970) Leading Article, i, 381.

EDINGTON, G. M. AND GILLES, H. M.—(1969) ' Pathology in the Tropics '. London (Arnold) p. 507.

EDINGTON, G. M. AND MACLEAN, C. M. U. —(1965) *Br. J. Cancer*, **19**, 471.

EISNER, G. M. AND LEVITT, M. F.—(1961) in ' Progress in Liver Diseases '. Edited by H. Popper and F. Schaffner. London (Heinemann Med.). Vol. 1, p. 119.

FOLI, A. K., SHERLOCK, S. AND ADINOLFI, M.—(1969) *Lancet*, ii, 1267.

FOY, H., KONDI, A., PARKER, A. M., STANLEY, R. AND VENNING, C. D.—(1970) *Lancet*, i, 1336.

GITLIN, D. AND BOESMAN, M.—(1967) *Comp. Biochem. Physiol.*, **21**, 327.

GOLD, P.—(1967) *Cancer, N.Y.*, **20**, 1663.

GRAHAM, E. R. B.—(1966) in ' Glycoproteins. Their Composition, Structure and Function '. Edited by A. Gottschalf. Amsterdam (Elsevier) p. 352.

GRANT, G. H.—(1964) in ' Recent Advances in Clinical Pathology '. Edited by S. C. Dyke. Series IV. London (Churchill).

HIGGINSON, J.—(1956) *Br. J. Cancer*, **10**, 609.

HULL, E. W., CARBONE, P. P., GITLIN, D., O'GARA, R. W. AND KELLY, M. G.—(1969) *J. natn. Cancer Inst.*, **42**, 1035.

KITHIER, K., HOUŠTĚK, J., MASOPUST, J. AND RÁDL, J.—(1966) *Nature, Lond.*, **212**, 414.

LAMBOTTE, R., SALMON, J. AND LAMBERT, P. H.—(1964) ' Protides of the Biological Fluids '. Edited by H. Peeters. Amsterdam (Elsevier) Coloquium 12, p. 207.

MASOPUST, J., KITHIER, K., RADL, J., KOUTECHKY, J. AND KOTAL, L.—(1968) *Inst. J. Cancer*, **3**, 364.

MAWAS, C., BUFFE, D. AND BURTIN, P.—(1970) *Lancet*, i, 1292.

MORRIS, M. N., POWELL, S. J. AND ELDSON-DEW, R.—(1970) *Lancet*, i, 1362.

O'CONOR, G. T., TATARINOV, YU. S., ABELEV, G. I. AND URIEL, J.—(1970) *Cancer, N.Y.*, **25**, 1091.

PEDERSEN, K. O.—(1944) *Nature, Lond.*, **154**, 575.

SAKAGUCHI, H.—(1968) *Acta path. jap.*, **18**, 407.

SANKALE, M., SOW, A. M. AND BAO, O.—(1970) *Ghana med. J.*, **9**, 44.

SHERLOCK, S., FOX, R. A., NIAZI, S. P. AND SCHEUER, P. J.—(1970) *Lancet*, i, 1243.

STANISLAWSKI-BIRENCWAJG, M.—(1967) *Cancer Res.*, **27**, 1982.

TATARINOV, YU. S.—(1964) *Vop. med. Khim.*, **10**, 584. (Translated in *Fedn Proc. Fedn Am. Socs exp. Biol.*, 1965, **24**, T916.)—(1965) *Vop. med. Khim.*, **11**, 17. (Translated in *Fedn Proc. Fedn Am. Socs exp. Biol.*, 1966, **25**, T344.)

VAN FURTH, R. AND ADINOLFI, M.—(1969) *Nature, Lond.*, **222**, 1296.

ZÜHLKE, V. AND HERMANN, G.—(1969) *Z. ges. exp. Med.*, **149**, 333. (Translated abstract in *Germ. med. Mon.*, 1970, **25**, 175.)